# Traditional ecological knowledge on the slope of Mount Lawu, Indonesia: all about non-rice food security

**DOI:** 10.1186/s42779-022-00120-z

**Published:** 2022-03-07

**Authors:** Sumarwati Sumarwati

**Affiliations:** Applied Linguistics and Literature Research Group, Universitas Sebelas Maret, Surakarta, Central Java Indonesia

**Keywords:** Traditional ecological knowledge, Non-rice food security, Folktale, Ritual, Taboo, Sustainability

## Abstract

As a country with rice as the staple food, the dominant traditional ecological knowledge (TEK) on food in Indonesia is rice. However, in Tawangmangu District, Central Java Province, the TEK inherited from their ancestors is about non-rice food security. This study aimed to explore how villagers pass on and practice their knowledge and beliefs in food defense based on traditional ecological knowledge. The data were collected through FGD, interviews, and field observations of traditional ceremonial processions, agricultural activities, and natural resource management. The results showed that TEK in Tawangmangu had three themes, including (1) TEK communication and inheritance through folktales on the origin of vegetables and corn, taboo words, such as the prohibition of planting rice, and the symbolic meaning of traditional rituals and offerings; (2) the people’s philosophy is reflected in their view of God, ancestors’ spirits as folktales figures, village guards’ spirits, and other living things. The folktales protagonists’ spirits are asked to provide protection, while the antagonists' spirits are asked not to interfere; and (3) natural resources sustainability involves maintaining non-rice plant commodities, terraced agricultural land management, intercropping systems, managing water resource and crop yields, and traditional houses architecture. The locals protect the forest on Mount Lawu and Pringgodani Cave as their source of life by prohibiting cutting trees and reforestation.

## Introduction

An indigenous community has distinct social, economic, practical, spiritual, political, and historical ties to ancestral lands [1]. The relationship system is passed through generations as a guideline in various fields, including farming, irrigation, fishing, forest security, village cleanliness, and construction. The community’s local wisdom and cultural heritage result from their habits or culture to adapt to nature and the environment [2]. As a result, they develop, improve, and protect this knowledge for centuries by passing it through generations. The indigenous community’s environmental management methods and practices are based on traditional ecological knowledge or TEK [3].

TEK significantly contributes to the natural environment, ecology, biology, geology, and geography for sustainability [4]. Various countries have researched TEK focusing on resource sustainability and environmental conservation, such as biodiversity [5–7] and forest management [8]. However, some studies revealed that it is lacking in most countries, including China [9, 10], South India [11], and Fiji [12]. TEK has become extinct globally as past and oral traditions [13]. Since it strengthens people's resilience to manage various global changes, losing it will reduce the ability to manage climate change [5, 14]. As a result, UNESCO [15] and the World Intellectual Property Organization [16] urge countries to safeguard TEK sustainability.

Agea et al. [17] stated that TEK is essential in food production for developing countries. Studies in various countries indicate that the indigenous community has knowledge and practices to select food commodities and manage and process food crops. The community in Godda District, Jharkhand, India, has knowledge of diverse and nutritious traditional food, including the benefits of consumption or avoiding them, such as taste, availability, season, cost, and processing duration [18]. Following the indigenous knowledge passed through generations, Limpopo Province, South Africa, applies various indigenous of food processing methods and coping strategies during food shortages [19]. The community’s food production and preservation knowledge in Turkana, Kenya, improves food security as a resource-scarce country [20]. This knowledge is through continuous community interactions and environmental observations.

Indonesia has over 300 ethnic groups with unique traditional knowledge [21]. It is the fourth biggest agrarian developing country globally [22], with its TEK linked to food security. There are various traditional ecological knowledge studies on food issues. For example, research published in an Indonesian-language manuscript stated that a community in Gorontalo Province applies local wisdom for food independence without relying on a single food commodity and diversifies food sources besides rice [23]. Some Dayak communities in West Kalimantan Province apply a traditional rice farming system, namely a shifting cultivation system intercropped with other foods while maintaining abandoned forests [24]. Another research focused on the community around the forest of Tesso Nilo National Park at Riau Province, possessing traditional knowledge by prohibiting forest damage, essential for farmers unable to plant rice due to flooding in the rainy season [25]. A study on traditional knowledge application in rice cultivation by the Tomohon community, North Sulawesi Province, concluded that they use some traditional knowledge and abandon others, such as cooperation harvesting because machines replaced the harvesting system [26]. Additionally, the Moluccas Islands community is famous for their traditional planting and processing methods of sago as the staple food [27].

As a country whose primary food is rice, traditional knowledge in Indonesia is dominated by these foodstuffs. For example, based on the Dewi Sri folktale, the community in Java Island believed that God chose rice for the farmers as the only grown plant [28]. Therefore, rice is their staple food, but some residents consume traditional non-rice foods. The local community’s traditional food is tubers in Papua and sago in Maluku. Furthermore, some groups consume traditional non-rice staple foods such as corn and tubers. This community lives on Mount Lawu slopes, especially Tawangmangu District, Karanganyar Regency, Central Java Province.

The community in the Tawangmangu District consumed corn and tubers as their staple food until early 1990. They did not consume rice like most Indonesians due to the area's climatic and geographical conditions, especially high rainfall and steep slopes, unsuitable for rice cultivation [29]. However, food self-sufficiency programs promote the residents consuming non-rice to include rice as a main source of nutrition gradually. Indonesia implemented food security policies in the 90 s, focusing on single food production, with rice as the staple food termed ‘rice self-sufficiency’ [30, 31]. This caused a transformation from diverse to uniform staple food, where the community consuming non-rice staple food switched to rice. Therefore, non-rice staple foods such as corn, tubers, and sago lost their capacity in food defense [32]. As a result, the consumption of corn as a staple food was abandoned after the 90 s. Some Javanese do not feel full or considered ‘have not eaten’ without eating rice. Corn rice and cakes are offered during the Dukutan and Mondosiyo village's clean traditional ceremony.

Despite corn being a non-staple food in Tawangmangu, TEK on non-rice food still exists, including folktales on the origin of non-rice commodity crops, traditional ceremonies with non-rice offerings, and agricultural systems for non-rice crop management [29, 33]. Most residents' lives revolve around non-rice food security, believing that the folktale events on the origins of corn and vegetables are real. Following this belief, the resident plant selected commodities, disregarding local climatic and geographical conditions, unsuitable for rice cultivation. Furthermore, their beliefs are demonstrated in daily life and traditional ceremonies [33].

Huambachano [34] viewed that the indigenous community’s food is related to local TEK; hence, food shifts reduce TEK. That is because TEK is special and unique so it applies to certain areas and is different from other areas [35]. TEK includes knowledge, practice, and beliefs based on food issues maintenance, such as the origin of food, farming and harvesting procedures, pests’ control, harvest management, and disasters management [20]. Communities on Selaru Island, Maluku Province, have local wisdom on planting, harvesting, and processing ways for various non-rice commodities. However, the rotation is reduced because food commodities are abandoned and replaced with rice [36]. The shift in staple food for the Tawangmangu District community allows a shift in their traditional ecological knowledge, especially knowledge, beliefs, and practices related to its maintenance. However, their defense practices remain despite losing TEK knowledge and beliefs. This is similar to Sri Lanka with a unique native food tradition, but now the dissemination of the knowledge is limited due to the decrease of the number of people having the knowledge [37].

Various TEK assets in Indonesia are near extinct, such as knowledge on shade trees selection in nutmeg plantations in Ambon (Maluku Province) due to the lack of local authorities’ support [38] and biodiversity conservation knowledge by the Dayak tribe in North Kalimantan Province because the younger generation does not care [39]. The Dayak community in East Kalimantan Province has TEK on selecting a suitable land plot, soil classification, and 'signs from nature' embedded as a culture—requiring focus during certain months and types of activities. However, this knowledge is near extinct due to a lack of regeneration [24].

This research focused on why and how the Tawangmangu community inherits decision-making knowledge for selecting certain food crops and cropping patterns based on their traditional knowledge, including sustainability management. The results can help formulate policies on Tawangmangu District development as a tourism area. Furthermore, strategies mapping to maintain TEK continuity for the Tawangmangu community can reference non-rice heritage preservation considering Indonesia struggles to provide rice and should build a culture of diverse food consumption [40]. Therefore, this study explored the communities’ TEK in non-rice food security.

## Methodology

This was a case study that examined the real causality or cause and effect explanations in the study subject [41], focusing on ‘how’ or ‘why’ TEK questions. The selection of this approach was based on the intentions to reveal food security issues in Tawangmangu, especially on an individual or a social unit based on the existing conditions and contexts. The data were collected using various techniques, including field observations with the researcher as the main instrument, emphasizing the process and meaning from the subject’s perspective. This case study was contemporary and related to current and past conditions that affected the research period.

**Fig. 1:**
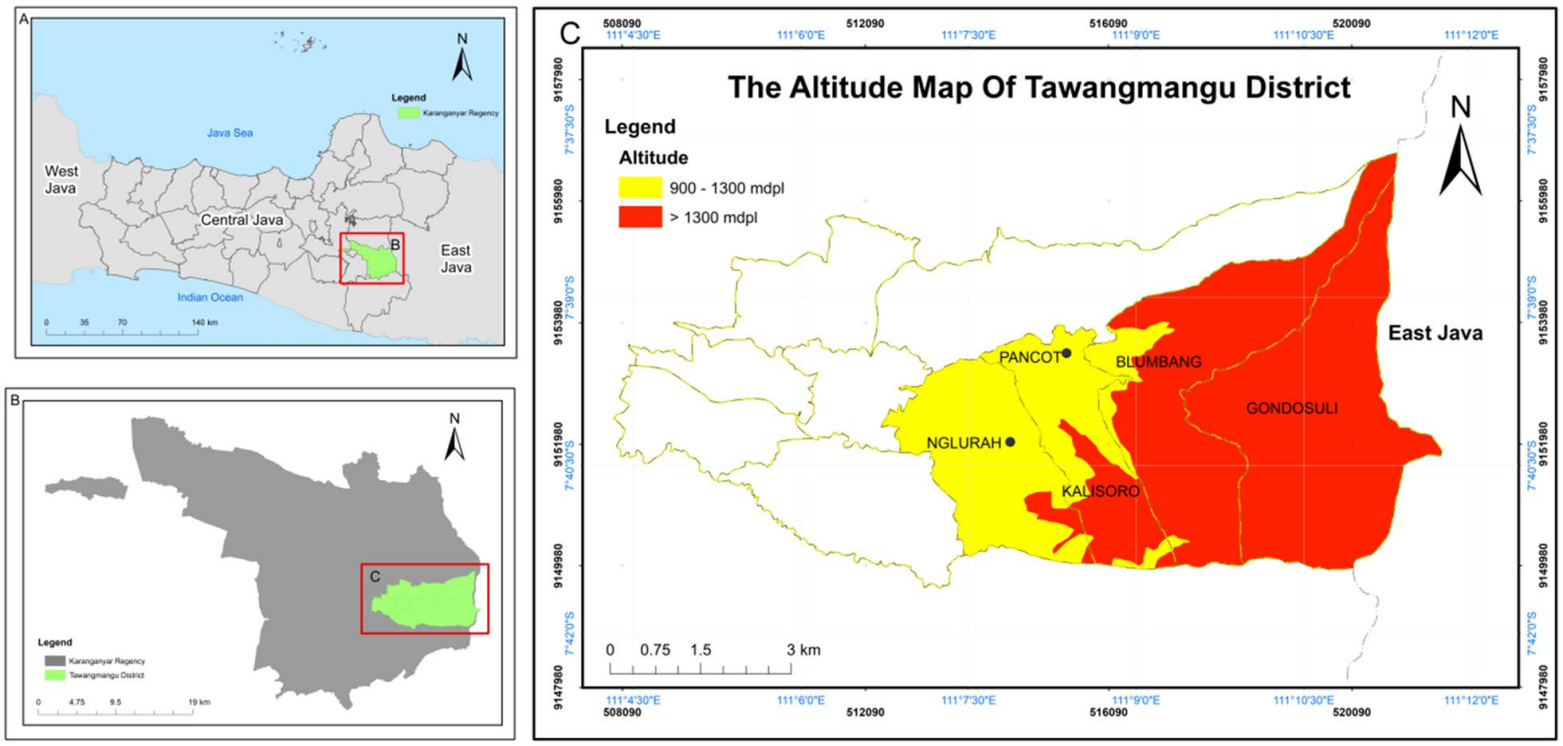
**A** Geographic location of Karanganyar Regency in Central Java Province, **B** Tawangmangu District in Karanganyar Regency, **C** Study area

The study was conducted in five villages on the Mount Lawu slopes, including Nglurah, Pancot, Kalisoro, Blumbang, and Gondosuli in Tawangmangu District, Karanganyar Regency, Central Java Province. Five out of ten villages were selected because the communities applied TEK. Tawangmangu is a popular tourist area due to its beautiful nature with Grojogan Sewu Waterfall. It is famous for vegetable production with a highland area of 1200 m above sea level altitude (Fig. 1). Furthermore, it is a steep hilly area with an elevation of 400–2200 m above sea level and a slope between 5° and 45°. It is a cold area even in the rainy season with 18 °C and receives the highest rainfall than other areas in Karanganyar Regency, even during the dry season.

Following the study objectives, the data collection focused on the TEK potential on non-rice food security in Tawangmangu. The sources included informants, the offerings making process, traditional ceremony procession, farming fields, planting and harvesting processes, traditional ceremony venue, Menggung Site, Pringgodani Cave, and traditional houses. The informants consisted of 4 TEK experts, the village leader, the local culture lovers’ group, offerings maker, senior farmers, and irrigation officer. 'TEK experts' include the indigenous people caring for the local culture to improve their knowledge than others. They were aged 55 to 75, often acting as the resource person for locals and outsiders, and one graduated in art and culture.

**Fig. 2:**
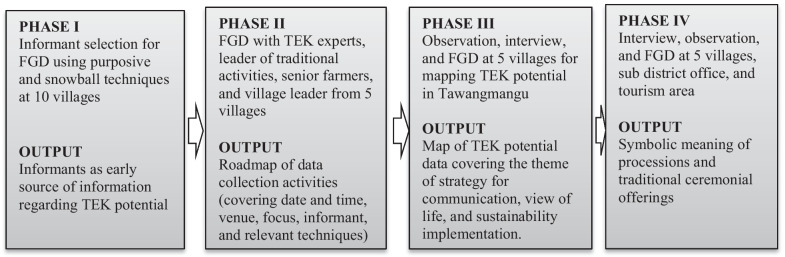
Phase of data collection

The data were collected through focus group discussions (FGD), observations, and in-depth interviews (Fig. 2). FGD was applied to plan the data collection strategy during the early phases, uncover folktale, interpret data, classify, and determine the TEK changes. The observations was focused around the offerings making process, traditional ceremony procession, farming system, reforestation, environmental preservation system, traditional houses characteristics, and TEK relevant areas. The interview was done to explore folktale, traditional ceremony procession and meanings, religious behaviors, views on unseen power, and farming system.

This study explored the traditional knowledge in Tawangmangu, focusing on three themes by Turner, Ignace, & Ignace [1], namely communication and knowledge exchange, philosophy and worldview, and TEK defense practices and strategies.

## Results

### Potential of traditional ecological knowledge in Tawangmangu

**Fig. 3:**
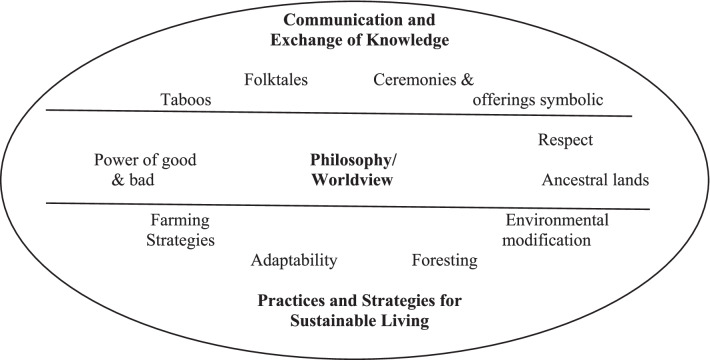
Map of TEK in Tawangmangu

TEK data in Tawangmangu can be classified into three themes. Figure 3 exhibits the details as the following.

### Communication and exchange of knowledge

The ecological knowledge on non-rice food security is passed through generations verbally and non-verbally. Verbal communication includes folktale and taboo statements, while nonverbal includes symbolic meanings in traditional ceremonial processions and offerings.

### The folktale of the origin of non-rice food

There are two folktales believed to be true. *The first* is the Narotama folktale that conveys the origin of the corn in Nglurah Village. In the ancient times, King Airlangga, a warlord named Narotama, and his soldiers fled because enemies attacked the kingdom in ancient times. They rested in Nglurah village along the journey, and Narotama decided to stay because many residents lacked food. The residents had poor rice harvest; hence, he introduced them to corn suitable for high rainfall and steep slopes. He taught the residents how to process corn into a staple food, namely corn rice. Therefore, corn became the staple food in Nglurah Village and other villages in Tawangmangu, and the residents no longer lacked food or died of starvation. Narotama was known as Kyai Menggung in his later years, buried in his former residence, and his tomb named the Menggung Site.

*The second* folktale is titled King Baka and Putut Tetuka, with the character being a king named King Baka as a man-eating giant. The residents offered their family members to be eaten, and the king’s cruelty added to their suffering, including starvation due to failed rice crops. A hermit named Putut Tetuka replaced an old widow when the king wanted to eat him. Therefore, his supernatural powers prevented the king from cutting his body into pieces and was swallowed whole. Putut Tetuka jumped from the king's stomach, causing a war between the two for days. King Baka was defeated and died by hitting his head on a black rock. He realized his cruelty before dying, and to atone for his sins; he said, ‘O gods, I have made my people suffer, as a penance for sins, I beg you to transform my body into the earth’s produce so that my people do not lack food.’ His body parts turned into crops such as bananas, cassava, potatoes, and others. Before his imprisonment in a cave, Putut Tetuka advised the residents to plant vegetables or secondary crops and forbade planting rice. Furthermore, it is unknown when Putut Tetuka was nicknamed Kyai Kacanegara.

### Non-rice food taboo

The resident needs to follow several prohibitions based on efforts to maintain non-rice food. *First,* the ‘ban on planting rice’ in Tawangmangu followed, by farmers who believe that a disaster will occur when they disobey. Some farmers stated that the landslides and disease outbreaks were due to planting rice during their ancestral period. *Second*, ‘the prohibition of making non-corn offerings’ allowing only corn complements during traditional ceremonies. This is based on the belief that Narotama and Putut Tetuka requested corn offering to commemorate their services. Violating the prohibition is believed to cause natural disasters or vegetable and secondary crops failure. *Third*, ‘the prohibition of tasting corn dishes offerings’ because they are offered to respected ancestors (Kyai Menggung and Kyai Kacanegara), prohibiting tasting or opening the pot. The offerings are provided at the Dukutan and Mondosiyo village clean traditional ceremonies. It is believed that violating the prohibition causes uncooked food. *Fourth*, ‘banning making corn offerings by unholy women,’ prohibiting tasting and corn rice cones preparations by menstruating women with washed hair. This is because the offerings are for sacred ancestors and must be made by holy people.

### The symbolic meaning of the traditional ceremonial procession

Communities on Mount Lawu slopes hold various ceremonies, including the traditional ceremony of village clean Dukutan and Mondosiyo and Dawuhan water security. It is a large traditional ceremony involving all residents every 7 months, believing in disaster when the ceremony is ignored.

The village clean Dukutan ceremony held in Nglurah Village honors the services of Narotama or Kyai Menggung. The process involves: *First*, the residents clean the Menggung Site, waterways and agricultural land, village, and the *pendapa* 'the ceremony area,’ a day before the event. They believe that Menggung is the residence and tomb of Kyai Menggung and his wife; hence, the procession symbolizes ‘keeping a clean physical and mental environment.’ *Second*, the residents collect corn rice cones and side dishes and pray at the village *pendapa*, symbolizing ‘gratefulness and praying for a bountiful harvest.’ *Third,* the procession surrounds the village with corn offerings and *palawija* ‘second crops,’ cheering and performing *barongsai* ‘a lion dance’ to the Menggung Site. This symbolizes ‘rejoicing and enjoying the harvest.’ *Fourth*, the residents and visitors hold a ‘war’ brawl by throwing corn rice and apologizing to each other, commemorating the war between Kyai Menggung and Nyai Menggung before getting married. The war was caused by Nyai Menggung and her students who refused to plant corn; hence, the procession symbolizes ‘not to worry about selecting corn as a staple food.’

The Mondosiyo traditional ceremony is held in Pancot, Kalisoro, and Blumbang village. The procession includes: *First*, the residents clean the village, ancestor’s graves, Pringgodani Cave, and waterways a day before the event. They believe that Pringgodani Cave is where Putut Tetuka was meditated. The procession symbolizes ‘maintaining a clean physical and non-physical environment’*. Second*, they collect offerings at the village *pendapa*, praying and eating together with *gamelan* music, symbolizing ‘gratefulness for God’s gifts and the ancestors’ service.’ *Third*, the residents slaughter the *kendit* goat 'with black hair and a white belly, which is rare and expensive. This symbolizes ‘giving the best to the ancestors (others).’ *Fourth*, the residents wash the black stone that killed King Baka with sticky rice water (fermented glutinous rice) to relieve his pain. This symbolizes ‘empathy to those suffering’*. Fifth*, the *Reog Pancot* attraction accompanies the procession around the village, symbolizing ‘celebration and enjoying a bountiful harvest.’

Guarding the Dawuhan water ceremony occurs a month before the Dukutan ceremony. The residents believe that their ancestors passed the Dawuhan tradition to protect and preserve the springs. The villages with a water source participate in the water-protection ceremony, gathering in the water source area for offerings, cleaning and preventing environmental damage, and praying and eating together. This symbolizes ‘gratefulness for the gift of continuous water flow through the agricultural land.’

### The symbolic meaning of traditional ceremonial offerings

As stated by a local TEK expert, all the traditional ceremonies include offerings made from corn for rice and cakes. The residents are prohibited from using rice, and it takes two days to make corn offerings. It is soaked for two nights before turning into flour then pounded in a mortar. However, since the community familiarized with the grinding tool, they take it to the mill for a smoother texture and save energy. The corn is ground into fine flour, mixed with grated coconut seasoned with an estimated amount of salt, which prohibited to be tasted, and steamed until half cooked. This mixture is divided by two, with one part steamed to make corn rice while the other makes cakes. The other foodstuffs provided include cassava (*Manihot esculenta*), sweet potato, and banana (Musa). The side dishes provided must also come from the environment around the village, for example tempeh 'fermented soybean (Glycine max) and *ares* 'banana stem hump (Caulis)'. The scientific names of the offerings and their equipment can be read in Table 1.

**Table 1:** List of scientific and native names of material offerings

Scientific name	Native name	Utilization
*Zea mays*	*jagung*	*Jagung* ‘corn’ for making corn rice and cakes. The process is as follows: the cornstarch is mixed with shredded coconut and a little bit of salt, then kneaded, before steaming for 30 min. The dough is then split into 2 parts. One part is steamed for another 30 min to make corn rice. To make the *tumpeng* ‘the cone-shaped yellow rice’, the corn rice can be moulded into a cone shape. To make the cakes, the dough can be molded into multiple shapes (*gandik*, *lingga*, human statues, etc.), to be steamed again for another 30–45 min
*Midrib*	*pelepah*	The *pelepah* ‘banana midrib’ for making the *encek* ‘tray’. The making process is as follows: The midrib is shaped into rectangles which are connected by sticks made from bamboo (Bambuseae). The top part is then covered with banana leaf (Leaf blade) as a lining
*Glycine max*	*kedelai*	Soy is used to make tempeh and *bongko*. The process of making tempeh is as follows: soy is washed and soaked for one night, peeled, steamed for 45 min, and then fermented for 2 to 3 days. As for *bongko*, black soy is used. The black soy is soaked for 1 night, mashed, seasoned, wrapped with banana leaf, and then steamed for 30 min
*Musa*	*ares*	The part of the banana plant used is *ares* ‘the innermost part of the banana trunk’. The making process is as follows: *ares* is cut into cubes, boiled for 30 min, seasoned, wrapped with banana leaf, and steamed for 30 min
*Artocarpus camansi*	*kluwih*	The *kluwih* fruit is used to make *lodeh* dish 'coconut milk vegetable dish'. The following is the process making: peel the fruit, cut it into cubes, boil, give seasoning, and mix with coconut milk
*Lpomoea batatas*	*ubi*	*Ubi* ‘sweet potatoes’ are used to make *kolak* *ubi* (sweet potatoes with coconut milk) and boiled sweet potatoes. The process of making *kolak ubi *is as follows: cut sweet potatoes into cubes, boil, and then mixwith coconut milk. The process of making boiled sweet potatoes is as follows: the clean sweet potatoes and then steam them

**Fig. 4:**
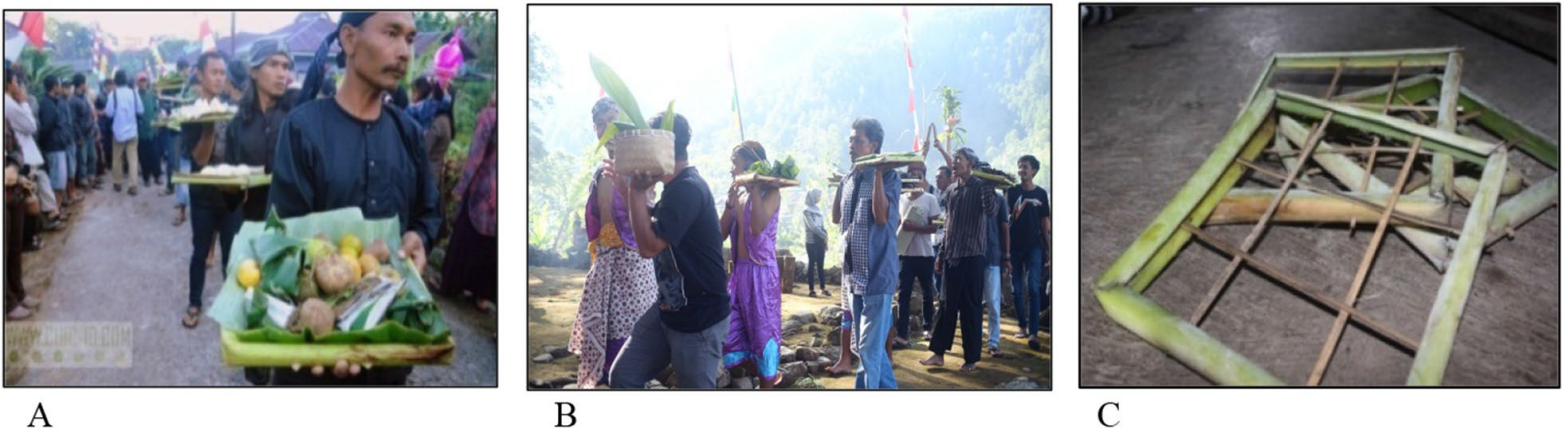
**A**, **B** Offerings brought on the procession on a tray called *encek*; **C** *Encek*. *Source*: author

The offerings area is made from common material from the village, namely the banana leaves midrib as shown in Fig. 4.

**Fig. 5:**
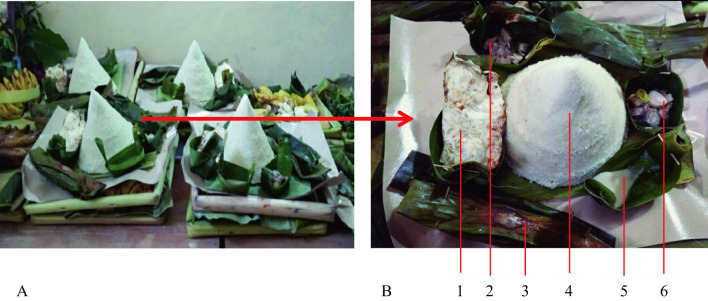
**A** Offerings sent by each family. **B** Contents of offerings: 1. grilled *tempe* ‘fermentation soya’; 2. *kolak ubi* 'sweet potato cooked with coconut milk,' 3. *botok ares* ‘a side dish of banana hump (*Caulis*) which is seasoned and wrapped in leaves and then steamed'; 4. *tumpeng nasi jagung* 'cone-shaped corn rice'; 5. coconut milk; 6. *kluwih* soup (*Artocarpus camansi*) ‘cooked with spices and coconut milk’ and a half of boiled egg. *Source*: author

The traditional ceremonies in Tawangmangu have a similar type of offerings. The main offering is corn rice cone 'conical rice corn' and its complements (Fig. 5). The offering maker stated that the corn rice is shaped like a cone with a top central point and the side dishes symbolize the mountain and ecosystem. A mountain is sacred for the Javanese because it is close to the sky and heaven. The side dishes and vegetables are made from simple processed ingredients, such as baked tempeh without frying or seasoning, meaning that ‘people must live a simpler life than their ancestors.’

**Fig. 6:**
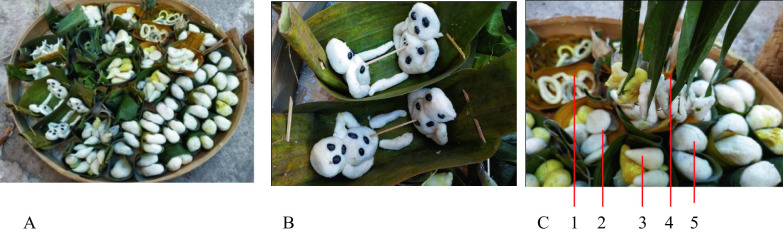
**A** Corn cakes of various shapes served in bowls made of banana leaves and laid out on *tampah* 'trays of woven bamboo'; **B** Cake in the shape of a pair of people; **C** Pairs of cakes that resemble: 1. bracelets, 2. coins, 3. phallus, 4. necklace, and 5. *gandik* 'yoni.' *Source*: author

Another offering is a corn cake with different shapes between Mondosiyo and Dukutan ceremonies. The Mondosiyo ceremony has various shaped cakes, such as a pair of human statues, *gandik* 'yoni,’ *lingga* 'phallus,’ yoni, bracelets, necklaces, money, and bamboo clumps. Furthermore, each unique shape is made as a pair, symbolizing ‘that all the global phenomena occur in pairs,’ for example, old—young, sad—happy, rich-poor, healthy—sick. This symbolizes ‘that people must remember the times when young, healthy, rich, or happiness will not last forever.’ On the other hand, people should not give up when sick, poor, or difficult. The *gandik* symbolizes ‘women's womb or fertility goddess,’ while the *lingga* of humans symbolizes ‘males or source of seed’ as the source of life. Furthermore, cakes shaped as money, bracelets, and necklaces symbolize ‘human wealth or wrathful lust.’ Each uniquely shaped cake comes in three colors, white, yellow, and black. The yellow color uses turmeric water, while soot is used for black. The white pays ‘purity,’ yellow brass ‘majesty,’ while black belongs to ‘purity.’ The offerings are placed in a bowl made of banana leaves arranged on a 'woven bamboo tray' (Fig. 6).

**Fig. 7:**
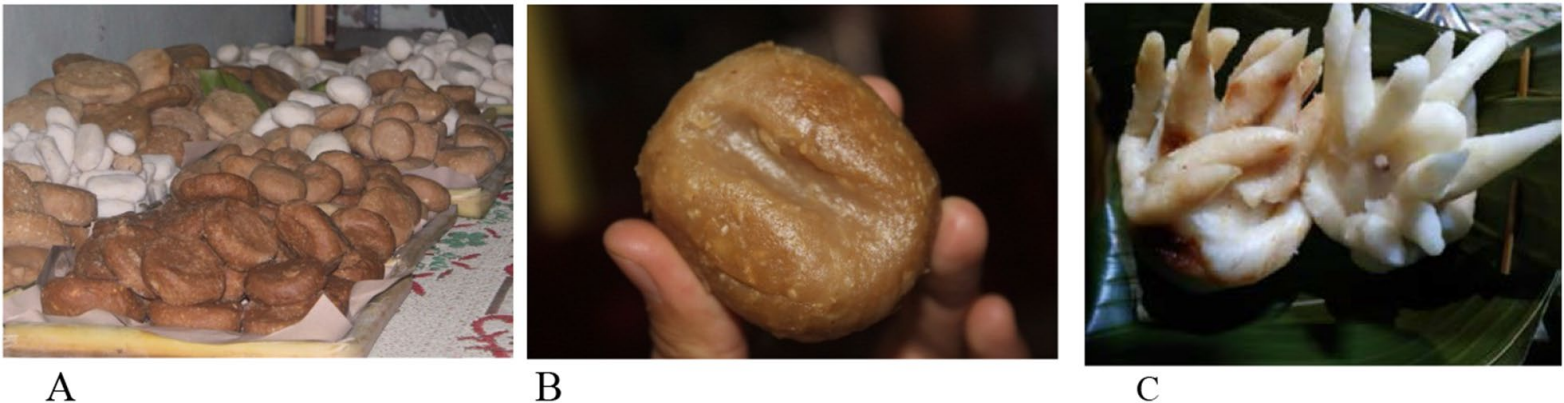
**A** *Gandik* 'cake made from corn' is red and white made from corn flour, grated coconut and a little salt. After the ingredients are mixed, they are shaped and steamed. **B** The form of *gandik* 'yoni.' **C** A Bamboo clump cake with a combination of red and white. *Source*: author

The Dukutan ceremony corn cakes in Nglurah Village are similar to the Mondosiyo traditional ceremony in Pancot, Blumbang, and Kalisoro villages. However, the cakes in Nglurah Village have two colors, red and white, made from mashed corn (Fig. 7). The red color includes melted brown sugar, symbolizing the female seed, while the white symbolizes the male seed uniting to birth a new life. The village clean ceremony of Dukutan uses a bamboo clump-shaped cake with red and white colors, symbolizing ‘a strong family kinship from everyone’s support and help’ (Fig. 7C).

**Fig. 8:**
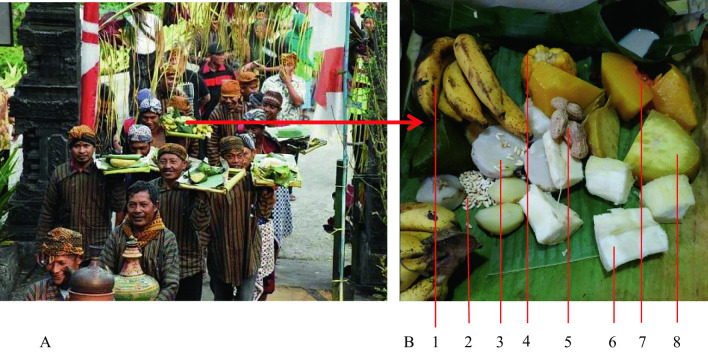
**A** Procession brings offerings of secondary crops 'non-rice food ingredients' (Photo retrieved from https://www.krjogja.com/berita-lokal/jateng/solo/upacara-adat-dhukutan-wujud-penghormatan-leluhur/); **B** The offerings include: 1. banana, 2. pumpkin seeds, 3. boiled white sweet potato, 4. boiled corn, 5. boiled peanuts, 6. boiled cassava, 7. boiled pumpkin, and 8. boiled yellow sweet potato. *Source*: author

Each family provides boiled bananas, corn, sweet potatoes, cassava, pumpkin, and peanuts offerings (Fig. 8). Boiled *palawija* ‘secondary crops’ symbolizes ‘humans earth offerings’ that they should treat the earth better for good results. Some of the offerings are consumed together, while others are brought during the village procession.

**Fig. 9:**
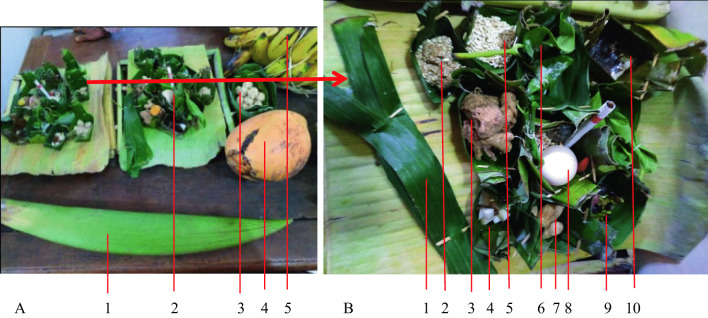
**A** Offerings placed in the neighborhood coordinator's house include: 1 coconut flowers, 2 *uba rampe* 'offering utensils,' 3 *tawonan kecil* 'corn cakes in small round shape,' 4 young coconut, 5 bananas. **B** The contents of the offerings are 1 wrapped roses, 2 *ares* ‘spiced banana stem,’ 3 ginger, 4 bananas with coconut milk sauce, 5 skinless peanuts, 6 betel leaf and whiting, 7 boiled peanuts, 8 boiled eggs, 9 fried black soybeans, 10 cooked coconut milk. *Source*: author

The *uba rampe* or 'set of offerings' is held on a Monday, a day before the event. The offerings are kept at the neighborhood coordinator’s home (village head) with the corn rice cone instead of the ceremony or procession area. It consists of young coconuts and flower buds, bananas, small round corn cakes, and other utensils, symbolizing good teachings (Fig. 9). In Javanese, a young coconut is called *cengkir*, associated with the term *kenceng ing pikir*, 'strong thinking,’ meaning that humans have strong thoughts with strong prayers and aspirations. *Mancung* ‘a sword-shaped coconut bud’ symbolizes self-confidence, meaning that humans navigate life with confidence, belief and have faith in God Almighty. A pair of bananas (banana king) symbolizes human ideals, meaning that ‘determining ideals follows good ways, bringing safety, prosperity, and happiness to the nation and universe.’ Betel (*Piper betle L*), areca nut (*Areca catechu L*.), and whiting (*Calcium hydroxide*) symbolize respect, meaning ‘we must respect each other to live in harmony and peace.’ This is based on the ancient Javanese tradition when welcoming guests with a set of ‘betel-areca nut-whiting,’ signaling respect.

### Philosophies and worldviews

#### Belief in the power of good and bad

Based on supernatural entities, the residents believe in two powers and strengths, good and bad. The good entities include Kyai Menggung (Narotama), Kyai Kacanegara (Putut Tetuka), and Eyang Nata Kusuma (the ancestral spirits guarding the village). The bad ones include King Baka and other creatures considered as plant nuisances. They ask for safety and prosperity against good power and strength and lack of interference and damage from the bad. Therefore, citizens respect both but believe that God has the greatest power.

The Dukutan and Mondosiyo traditional ceremonies and offerings reflect trust in the above figures, including the origin of corn (Kyai Menggung) and vegetable folktales (Kyai Kacanegara and Raja Baka). The residents hold certain ceremonies to protect the village and the environment from disaster based on their beliefs. Therefore, they conduct timely and effective village cleaning ceremonies, even during the COVID-19 pandemic. The community associates a disaster with deficiencies in the traditional ceremonies implementation believe that the high fatalities in the past epidemics of deadly disease and erosion were due to a lack of traditional ceremonies.

#### Respect for all entities

The Tawangmangu community maintains its cultural norms like other indigenous people living in balance and harmony with nature. The locals view the environment as a whole, with all parts interconnected in a network of causes and effects, actions and outcomes, and behaviors and consequences. There is an interactive relationship between humans, plants, natural objects, and supernatural entities, inseparable and distinct relating to each other. Therefore, humans must rightfully treat other creatures, including plants and animals. A local senior citizen stated that they sought the tree keeper’s permission before cutting trees. Furthermore, they cannot get rid of ants in the house and instead put sugar or food to move and focus on the behavior and character of other entities as natural phenomena are associated with human life. For example, people who find a four-leaf clover (*Salviniales*) are filled with good luck and select the plants based on the belief that vegetables and secondary crops are the ancestors' choice to be preserved through generations.

#### A Belief in sacred places

Most people believe in the myths of folktale characters, giving meaning to the ceremonies. This is based on the belief that the folktale of Narotama and Putut Tetuka is true. Therefore, they believe certain places belong to these characters, including the Menggung Site in Nglurah Village. It has a large tree (tree circumference ± 10 m) thought to be centuries old, and the residents are forbidden to cut the tree. The Menggung site is a sacred place believed to be Narotama’s and his wife’s residence and burial place (Kyai Menggung), a folktale character on the origin of corn. Dukutan traditional ceremonial procession is held at the Menggung site. Second, the Pringgodani Cave on Mount Lawu slopes is sacred because it is believed to be the area to meditate Putut Tetuka (Kyai Kacanegara), a folktale character on the origin of plants. The residents are prohibited from cutting trees and damaging the sacred environment in the Pringgodani Cave area.

### Practices and strategies for sustainable living

#### Farming strategies

The farmers’ agricultural system in Tawangmangu includes; *First*, vegetables and secondary crops as the primary. The vegetables include leeks, lettuce, carrots, onions, garlic, beans, cabbage, and beans, while the crops include corn, sweet potatoes, cassava, and bananas. The plant selection is based on Dukutan and Mondosiyo myths, instead of cold climate and high rainfall characteristics. Besides vegetables and secondary crops, some Tawangmangu residents, especially the Nglurah area, work as ornamental plant farmers. However, most farmers grow vegetables and no rice. *Second*, planting occurs in the dry season because these plants can manage with less air. *Third*, the vegetables and secondary crops are grown through an intercropping system, namely planting several (two to four) plant commodities simultaneously. Senior farmers believe this system is the preferred economic and ecologic teaching. *Fourth*, the leek is the mainstay due to higher profitability than onion and garlic. This is because Tawangmangu has superior quality and expensive scallions than other regions.

#### Environmental modification

Following the hilly terrain, Tawangmangu’s agriculture adopts a terraced land system with a slope of 50°. Some farmers stated that it is difficult to water vegetables on a sloping than flat land, especially during the dry season. Despite the abundant water flow from Tawangmangu springs, vegetables require more water, especially during planting season, and must be watered daily. The springs reduce their water discharge during the dry season on Mount Lawu slopes. The local officials called *jogotirto* 'water guards' coordinate the water usage in each village or neighborhood to avoid conflicts. Irrigation during the dry season follows a *girik* 'water ration card' system, regulating the schedule and water allocation for farmers. According to the farmers, the bill is based on the land area and required amount of water. The money is used to build and repair the irrigation canals. Furthermore, the residents plant secondary crops requiring less water due to expensive fees.

Vegetable farming follows the intercropping system, planting two or more crop commodities simultaneously and land. According to farmers, the intercropping method is conducted on plants with one harvest season, such as leeks with shallots and mustard greens. Planting various kinds of vegetables in one area prevents pests, anticipates reduced prices for certain vegetables, and meets the family needs. Furthermore, a Farmer's Group was formed to improve their welfare. The group forms the members’ mindset to work together for better profits and quality, competing with imported vegetables in the market.

#### Adaptability

**Fig. 10:**
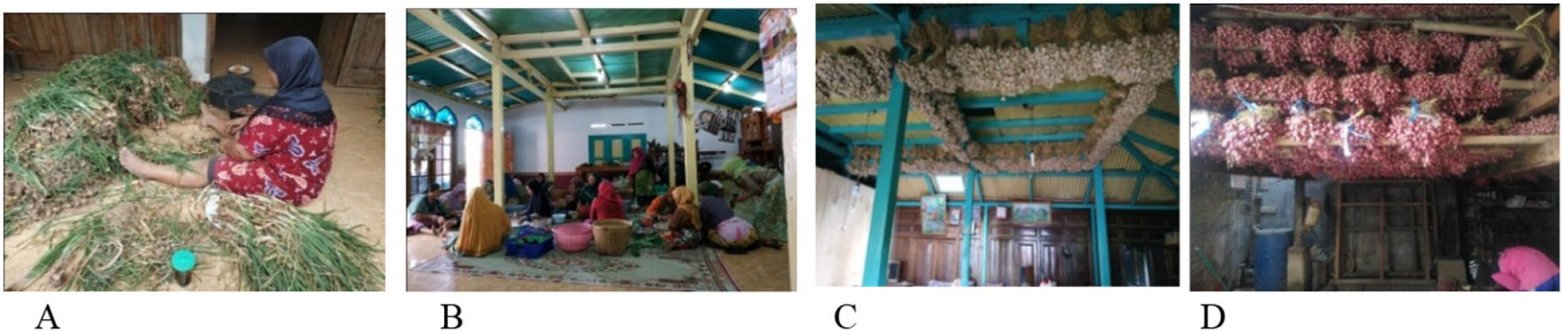
**A** Arranging shallot yields on the terrace; **B** making offerings together in the front room; **C** storing the garlic harvest on the roof of the vestibule; **D** storing the shallot harvest on the stove in the back room. *Source*: author

Traditional houses construction in Tawangmangu is affected by the residents’ ecological conditions, economic, and social life. The cold climate prevents the construction of traditional high houses, with tin roofing, without vents, and wood walls and pillars. Most traditional houses in Tawangmangu have large yards for planting vegetables and secondary crops or plants drying. Additionally, they have a terrace to store or clean crops. The traditional Javanese houses have three separate areas, the front, middle, and back (Fig. 10). The front room sits with the guests and conducts traditional activities, such as processing offerings in cooperation. Furthermore, they store the garlic harvest on the roof, and the middle room acts as the dining and bedroom area, while the back room holds crops, agricultural tools, kitchen, and bathroom.

#### Foresting

The community preserves the Slope Forest Area of Mount Lawu, Pringgodani Cave, and Ceto Temple to protect the environment and natural resources, especially their source of life sustainability, namely vegetable and secondary crops. According to local TEK experts and senior farmers, the Tawangmangu forest provides water sources and protects the ecosystem. Deforestation causes landslides due to hilly areas and dries the springs on mountain slopes. The ancient residents enforced customary rules on forest management around Mount Lawu. *First*, they required the environmental coordinators and village elders’ approval before cutting trees. *Second*, they planted replacement trees. *Third,* they held a ritual to seek the forest guardian spirits’ permission before cutting trees. *Fourth,* they conducted a tree-planting procession at the clean village ceremony.

## Discussion

Based on previous studies [1], TEK in Tawangmangu at the Mount Lawu slopes includes communication strategies, philosophy and way of life, and sustainability. TEK communication and passing is through two folktales on the origin of vegetables and corn, taboo statements (prohibiting planting rice), symbolic messages in the traditional ceremonial processions to respect the ancestors who introduced corn and vegetables as staple foods using corn offerings. The ancestral teachings on food security are passed through myths related to folktale characters. The residents believe these characters as entities with the power and strength of good and bad, respecting and placing them as humans with a resident sacred place. Following their beliefs, the residents conduct practices mandated by their ancestral such as food preservation, farming, managing crops, and sustainability. Moreover, they select palawija and vegetables as crop commodities disregarding the areas’ climate and geographical conditions but based on the Dukutan and Mondosiyo myths. The sustainability strategies of the local ecosystem include planting crops following an intercropping system, optimizing house functions, and forest preservation.

The findings were similar to previous researchers that TEK in rural areas is dominated by food security. For example, TEK in Turkana, Kenya, is related to food security [20], similar to Andes Mountains communities [42]. However, TEK in Tawangmangu follows one theme, non-rice food security. This is based on the two folktales allowing farmers to plant vegetables and pulses instead of rice, disregarding climatic and geographical conditions. Tawangmangu District is located on the Mount Lawu slopes as a cold area with high rainfall, unsuitable for planting rice. Therefore, the folktales described several taboo statements, especially prohibiting planting rice. Breaking this rule will expose the residents to a deadly disease outbreak. Therefore, the findings differed from the TEK studies by two researchers on the country's staple food by focusing on alternative food ingredients.

The study found that TEK in Tawangmangu uses verbal communication strategy and inheritance on non-rice food security through generations. This is relevant to previous findings that oral traditions spread the ancestral cultural heritage. Therefore, cultural wealth is easily changed or lost. Sri Lanka has limited knowledge of indigenous and traditional foods due to changing lifestyles, fewer holders of traditional ecological knowledge, and flora and fauna resources [5, 37]. TEK food sustainability is essential to maintain good environmental management practices. Studies on Benin farmers (West Africa) showed that they conserve or plant trees to conduct traditional ceremonies [43]. Therefore, losing folktales on the origin of corn and vegetables in Tawangmangu hinders the next generation from recognizing the story’s important message, namely non-rice food security. The further consequence includes the non-applicability of good ancestral practices acquired through centuries.

Despite the indications that TEK is near extinct in Tawangmangu, this research showed that some indigenous communities maintain valuable teachings on environmental management. The maintenance strategies include TEK changes and adjustments with modernity, government policies, pests, and economic needs. This method maintains the local ecosystem sustainability to date, strengthening previous findings. Ugandans overcome various problems following ancestral teachings [44]. This indigenous knowledge manages challenges such as floods, droughts, diseases, and pest attacks. Ugandans often experience drought, developing the indigenous communities’ knowledge to predict and overcome such challenges. Transferring the previous generations’ knowledge and good practices are embedded in the culture through various rituals such as birth, initiation into adulthood, marriage, and death [45–47]. Furthermore, previous studies found that farmers applying TEK farming systems overcame the Indian crisis more successfully than those using modern systems [48]. Therefore, traditional ecological knowledge (TEK) is recognized as intellectual activities in various social, cultural, and environmental global contexts to sustain life over time [18, 49]. Most countries do not value TEK in natural resource analysis [47, 50]. Furthermore, the younger people showed less TEK understanding and appreciation [22, 46].

## Conclusion

The non-rice food defense technology in Tawangmangu has three major themes. *First*, TEK communication and inheritance is through stories on the origin of vegetables and corn, taboo words such as prohibiting planting rice, the symbolic processions meaning at village clean traditional ceremonies and protecting water sources, and corn offerings. *Second*, the philosophy or global view in Tawangmangu is reflected through God’s perspective, ancestral spirits as folktale characters, and other living entities. The folktale protagonists’ spirits protect, while the antagonists are asked not to interfere. Some people mutually respect certain plants and animals as humans. Furthermore, the residents should seek the tree spirit’s permission to take banana leaves and avoid killing animals like ants. *Third*, the natural resources sustainability practices and strategies include selection of crop commodities (non-rice food), terraced agricultural land management (in the irrigation system), vegetable cropping system (intercropping), water resources management (maintaining forest conservation), and harvest management (sorting crop commodities, cleaning, classifying, and storage). The locals protect the forest areas of Mount Lawu slopes and Pringgodani Cave as their source of life by prohibiting logging and reforestation.

## Data Availability

Not applicable.
